# Atorvastatin improves hyperlipidemia-induced erectile dysfunction via PKA-associated eNOS phosphorylation at the Ser633 site

**DOI:** 10.1093/sexmed/qfag094

**Published:** 2026-10-18

**Authors:** Xinghao Zhang, Xu Wang, Ziheng Zhang, Ziyang Han, Yang Sun, Xue Yang, Yun Chen

**Affiliations:** The First School of Clinical Medicine, Nanjing University of Chinese Medicine, Nanjing, Jiangsu 210023, China; The Affiliated Hospital of Nanjing University of Chinese Medicine, Jiangsu Province Hospital of Traditional Chinese Medicine, Nanjing, Jiangsu 210029, China; The First School of Clinical Medicine, Nanjing University of Chinese Medicine, Nanjing, Jiangsu 210023, China; The Affiliated Hospital of Nanjing University of Chinese Medicine, Jiangsu Province Hospital of Traditional Chinese Medicine, Nanjing, Jiangsu 210029, China; The First School of Clinical Medicine, Nanjing University of Chinese Medicine, Nanjing, Jiangsu 210023, China; The Affiliated Hospital of Nanjing University of Chinese Medicine, Jiangsu Province Hospital of Traditional Chinese Medicine, Nanjing, Jiangsu 210029, China; The First School of Clinical Medicine, Nanjing University of Chinese Medicine, Nanjing, Jiangsu 210023, China; The Affiliated Hospital of Nanjing University of Chinese Medicine, Jiangsu Province Hospital of Traditional Chinese Medicine, Nanjing, Jiangsu 210029, China; The First School of Clinical Medicine, Nanjing University of Chinese Medicine, Nanjing, Jiangsu 210023, China; The Affiliated Hospital of Nanjing University of Chinese Medicine, Jiangsu Province Hospital of Traditional Chinese Medicine, Nanjing, Jiangsu 210029, China; The Second School of Clinical Medicine, Nanjing University of Chinese Medicine, Nanjing, Jiangsu 210023, China; The First School of Clinical Medicine, Nanjing University of Chinese Medicine, Nanjing, Jiangsu 210023, China; The Affiliated Hospital of Nanjing University of Chinese Medicine, Jiangsu Province Hospital of Traditional Chinese Medicine, Nanjing, Jiangsu 210029, China

**Keywords:** hyperlipidemia-induced erectile dysfunction, atorvastatin, endothelial function, protein kinase A, endothelial nitric oxide synthase

## Abstract

**Introduction:**

Hyperlipidemia contributes to vasculogenic erectile dysfunction (ED) by reducing endothelial nitric oxide bioavailability. Although atorvastatin has been reported to improve ED, the upstream mechanism by which it restores endothelial nitric oxide synthase (eNOS) activity remains unclear. This study aimed to evaluate the therapeutic effect of atorvastatin in hyperlipidemia-induced erectile dysfunction (HLED) and to determine whether protein kinase A (PKA)-associated phosphorylation of eNOS at Ser633 contributes to this effect.

**Methods:**

Hyperlipidemia-induced erectile dysfunction was induced in rats by a high-fat diet. Animals received low- or high-dose atorvastatin, with tadalafil as a positive control. Erectile function, serum lipid profiles, oxidative stress, penile fibrosis, and cavernosal eNOS signaling were assessed. In parallel, oxidized low-density lipoprotein (ox-LDL)-injured rat corpus cavernosum endothelial cells (RCECs) were treated with atorvastatin in the presence or absence of the PKA inhibitor H89.

**Results:**

Atorvastatin improved dyslipidemia, reduced systemic oxidative stress, attenuated penile fibrosis, and partially restored erectile function in HLED rats. Hyperlipidemia-induced erectile dysfunction was associated with decreased eNOS phosphorylation at Ser1177 and Ser633 and increased phosphorylation at Thr495. Atorvastatin enhanced PKA activation and eNOS Ser633 phosphorylation while reducing inhibitory Thr495 phosphorylation in penile tissue. In ox-LDL-treated RCECs, atorvastatin reduced intracellular lipid and ROS accumulation and improved endothelial function, accompanied by increased PKA Thr197 and eNOS Ser633 phosphorylation. These effects were largely blunted by H89.

**Discussion:**

These findings suggest that atorvastatin ameliorates HLED by restoring endothelial function through a PKA-associated increase in eNOS Ser633 phosphorylation. This upstream endothelial mechanism may complement the action of phosphodiesterase type 5 inhibitors and provide additional benefit for hyperlipidemia-associated vasculogenic ED. The combined in vivo and in vitro design supports mechanistic relevance, although pathway inference relies partly on pharmacological inhibition with H89 and limited time-point analyses. Collectively, our results identify PKA-associated eNOS Ser633 activation as an important mechanism underlying the endothelial-protective effect of atorvastatin in HLED.

## Introduction

Erectile dysfunction (ED) is a prevalent disorder in males, clinically defined as the persistent inability to achieve or maintain penile erection sufficient for satisfactory sexual performance.^1^ While ED has multiple etiologies, vascular endothelial dysfunction is a major contributor to the condition. Indeed, ED and cardiovascular diseases (CVD) share numerous risk factors such as hyperlipidemia, hypertension, diabetes, smoking, and obesity, which all impair endothelial function.^2^ In particular, hyperlipidemia-induced atherosclerosis can lead to reduced endothelial nitric oxide (NO) synthase (eNOS) activity and NO bioavailability in penile arteries, resulting in insufficient cavernosal smooth muscle relaxation and erectile failure.^3^ Consequently, hyperlipidemia is recognized as an important risk factor for ED, and ED in such patients is often an early indicator of systemic atherosclerotic burden.^4^

Phosphodiesterase type 5 inhibitor (PDE5i) is the first-line therapy for ED, acting by enhancing the NO/cyclic guanosine monophosphate (cGMP) signaling pathway. However, their therapeutic efficacy critically depends on adequate endogenous NO production.^5^ For patients with pronounced endothelial dysfunction such as diabetes or hyperlipidemia, impaired upstream NO synthesis markedly limits PDE5i responsiveness.^6^ In these PDE5i non-responders, restoration of endothelial function and NO bioavailability represent a crucial therapeutic strategy.

Given these considerations, we focused on atorvastatin, a first-line therapy for hyperlipidemia and CVD. This agent exerts vascular effects beyond lipid lowering, including the enhancement of endothelial function and NO bioavailability.^7^ Since ED and CVD are increasingly regarded as clinical manifestations of systemic endothelial dysfunction, pharmacological strategies targeting vascular health in CVD may also benefit ED.^8^ Consistently, adjunctive statin therapy has been reported to restore responsiveness to PDE5i in patients with vasculogenic ED.^9^ This implies that its effect is realized through the recovery of endothelial function, not just symptomatic vasodilation.

Despite these observations, the exact mechanisms by which atorvastatin restores NO signaling in vasculogenic ED remain to be elucidated. Whether atorvastatin activates eNOS through alternative pathways under conditions of metabolic endothelial dysfunction is unclear. Notably, previous studies have shown that atorvastatin can enhance protein kinase A (PKA) activity,^10^ which plays a critical role in endothelial function and angiogenesis. Further evidence indicates that PKA-associated phosphorylation of eNOS at Ser633 is essential for sustaining NO production, particularly when classical eNOS activation pathways are impaired.^11^ Therefore, we hypothesized that atorvastatin improves hyperlipidemia-induced erectile dysfunction (HLED) via activation of the PKA/eNOS signaling pathway.

## Materials and methods

### Animals

Eight-week-old male Sprague–Dawley rats (SPF grade; Beijing Vital River Laboratory Animal Technology Co., China) were used. The animals were raised at a temperature of 20-25°C, relative humidity of 50%-60%, and artificial lighting with a 12-h light/dark cycle.

### HLED rats model and treatment

Prior to the experiment, male SD rats were screened using the apomorphine (APO) test to confirm normal erectile function. Rats were then randomly assigned to either the Control group or the high-fat diet group using a random number table. After 1 week of acclimatization, the Control group was fed a standard diet, which provided a physiological energy density of approximately 3.38 kcal/g, with 23.1% of energy derived from protein, 12.0% from fat, and 64.9% from carbohydrates. The high-fat diet was prepared using the same basal formulation (77.3%, w/w), supplemented with 10% lard, 10% egg yolk powder, 2% cholesterol, 0.5% sodium cholate, and 0.2% methylthiouracil. Methylthiouracil was added to facilitate the development of hypercholesterolemia, consistent with previous rat models.^12^ Both diets were obtained from Suzhou Shuangshi Laboratory Animal Feed Co., Ltd. (Suzhou, China).

After 8 weeks of dietary intervention, rats were fasted for 6 h with free access to water before blood collection. Blood samples were collected from the orbital sinus during the same light-phase time window, between 08:00 and 10:00. Serum was separated and used for lipid profile assessment. A second APO test was conducted, and rats that did not exhibit an erectile response were classified as successful HLED models. The successfully modeled rats were randomly divided into 4 groups using a random number table: HLED, low-dose atorvastatin (Atv-L, 1 mg/kg; Lipitor, USA), high-dose atorvastatin (Atv-H, 2 mg/kg), and Tadalafil (positive control, 2 mg/kg; Cialis, USA). Atorvastatin doses of 1 and 2 mg/kg were selected to approximate human-equivalent doses of 10 and 20 mg/day, respectively, for a 60-kg adult, based on body-surface-area normalization using the standard Km conversion.^13^ Tadalafil was administered at 2 mg/kg, corresponding to the clinical maximum dose of 20 mg/day by the same conversion.

A 4-week intervention followed. The Control and HLED groups were administered an equal volume of saline by daily gavage, while the Atv-L, Atv-H, and Tadalafil group received corresponding doses (dissolved in saline). During this period, the Control group continued on the standard diet, while the other groups remained on the high-fat diet. After the 4-week intervention, blood was again collected via the orbital sinus within the same fixed time window described above for further analysis, and the erectile function was re-assessed using the APO test. Intracavernosal pressure and mean arterial pressure (MAP) were measured under anesthesia prior to the final tissue harvest.

### Blood parameter detection

Blood glucose, high-density lipoprotein, low-density lipoprotein, total cholesterol (TC), and triglyceride levels were measured using an automatic biochemical analyzer (AU480, Beckman, USA). Additionally, serum levels of aspartate aminotransferase (AST; Nanjing Jiancheng, China), alanine aminotransferase (ALT; Nanjing Jiancheng, China), total bilirubin (TBIL; Solarbio, China), reactive oxygen species (ROS; CUSABIO, China), and oxidized low-density lipoprotein (ox-LDL; Nanjing Jiancheng, China) were measured using commercially available assay kits. Absorbance was measured using a microplate reader (Synergy HT, BioTek, USA) at the recommended wavelength.

### Erectile function assessment

Erectile function was evaluated using an APO (Sigma, USA)-induced erection test, intracavernosal pressure (ICP), and MAP measurements. For the APO test, rats were placed individually in transparent cages in a darkened observation room with minimal lighting for video recording. Apomorphine was administered subcutaneously at 80 μg/kg, after which each rat was continuously observed for 30 min.^14^ Erectile responses during this observation period were recorded using a digital camera (Nikon Z5, Nikon, Japan), and the number of erectile events was counted. An erection was defined as the visible protrusion of the penile glans, retraction of the foreskin, and penile engorgement.

Intracavernosal pressure and MAP were measured in rats under continuous isoflurane (RWD, China) inhalation anesthesia. Anesthesia was induced with 5% isoflurane and maintained with 1% isoflurane in oxygen at a flow rate of 1 L/min using an anesthetic machine (TAIJI-IE, RWD, China). The depth of anesthesia was monitored throughout the procedure by respiratory pattern, pedal withdrawal reflex, and hemodynamic stability. Physiological signals were continuously recorded using an RM6240E/EC multi-channel physiological signal acquisition and processing system equipped with software version E2.6 (Chengdu Instrument Factory, China).

For continuous MAP monitoring, the left common carotid artery was exposed through a cervical incision. The distal end of the artery was ligated, and the proximal end was temporarily clipped. A small V-shaped incision was made between the ligature and the clip, and a PE-50 catheter filled with heparinized saline was inserted toward the proximal end and connected to a pressure transducer. After the arterial clip was released and the arterial pressure waveform stabilized, MAP was continuously recorded throughout the ICP measurement procedure. A midline abdominal incision was then made to expose the pelvic cavity. The pelvic ganglion and cavernous nerve were identified by posterolateral dissection at the junction of the prostate and vas deferens. The prepuce was incised to fully expose the corpus cavernosum. A 25 G intravenous cannula prefilled with heparinized saline was inserted into the corpus cavernosum and connected to a pressure transducer for real-time ICP recording.

For erectile function assessment, the cavernous nerve was electrically stimulated using continuous monophasic square wave pulses at 5 V and 15 Hz. Each stimulation lasted 60 s, and the maximum ICP during each stimulation period was recorded as ICPmax. Both the left and right cavernous nerves were stimulated, with three stimulations performed per side. A minimum interval of 5 min was maintained between successive stimulations to allow hemodynamic recovery and prevent nerve fatigue.

For data analysis, the first stimulation on each side was excluded as an adaptation trial. The maximum intracavernosal pressure (ICPmax) values from the subsequent 2 stable stimulations on each side were averaged to obtain the side-specific ICPmax, and the bilateral values were then averaged to obtain the representative ICPmax for each rat. Stable MAP values obtained immediately before stimulation or during the stabilized inter-stimulation period were used for normalization. Erectile function was expressed as the ICPmax/MAP ratio.

Additionally, the levels of NO and cGMP in penile tissue were measured using commercially available assay kits (Mlbio, China). Absorbance was measured using a microplate reader (Synergy HT, BioTek, USA) at the recommended wavelength.

### Hematoxylin and eosin staining

Liver tissues were harvested, fixed in 4% paraformaldehyde, embedded in paraffin, and sectioned. The sections were deparaffinized, rehydrated through graded ethanol, stained with hematoxylin, differentiated, counterstained with eosin, dehydrated, and mounted. Histopathological changes were examined under a light microscope (ECLIPSE Ni-U, Nikon, Japan).

### Masson’s trichrome staining

Penile tissues were harvested, fixed in 4% paraformaldehyde, embedded in paraffin, and sectioned. Cross-sections were then stained with Masson’s trichrome to visualize collagen (blue) and smooth muscle (red). For each animal, 3 sections were analyzed. From each section, 5 non-overlapping fields were randomly selected under a light microscope (ECLIPSE Ni-U, Nikon, Japan). Collagen volume fraction and the smooth muscle/collagen area ratio in the corpus cavernosum were quantified using ImageJ software (version 1.51), serving as indices of fibrosis.

### Cell culture and treatment

Rat corpus cavernosum endothelial cells (RCECs) (RAT-iCell-f019, iCell, China) were cultured at 37°C in a humidified incubator with 5% CO₂ and maintained in Endothelial Cell Medium (ECM) (ScienCell, USA). When monolayers reached 70%-80% confluence, cells were detached with 0.25% trypsin (Gibco, USA) and passaged. After 2-3 passages, the cells were seeded into plates. According to the experimental design, they were divided into the following groups: Control group (Dulbecco’s modified Eagle’s medium [DMEM] only), ox-LDL group (DMEM +20 μg/mL ox-LDL), ox-LDL + Atv group (DMEM +20 μg/mL ox-LDL + 2 μM atorvastatin), and ox-LDL + Atv + H89 group (DMEM +20 μg/mL ox-LDL + 2 μM atorvastatin +10 μM H89).^10^^,^^15^ All experiments were conducted using cells within six passages.

### Cell viability assay

Cell viability was assessed with the Cell Counting Kit-8 (CCK-8) (Abbkine, China) in 3 sequential assays. Rat corpus cavernosum endothelial cells were cultured in 96-well plates (2 × 10^4^ cells/well) for 24 h. First, to evaluate the cytotoxicity of atorvastatin (Acmec, China), RCECs cultured in DMEM-high-glucose (Gibco, USA) were exposed to graded concentrations of atorvastatin (0.1, 0.5, 1.0, 1.5, 2.0, 2.5 μM) for 24 h.^16^ Second, to determine the optimal concentration of ox-LDL for establishing an endothelial dysfunction model, RCECs were exposed to a range of ox-LDL concentrations (0, 5, 10, 20, 50, 100 μg/mL) for 24 h. Cell viability was measured, and the concentration that induced a significant but non-lethal reduction in viability was selected for subsequent experiments. Third, to evaluate the protective effects of atorvastatin and identify the most effective concentration, RCECs were co-treated with the selected ox-LDL concentration (determined from the second assay) and graded concentrations of atorvastatin (same as above) for 24 h. For all assays, 10 μL of CCK-8 reagent was added to each well and incubated for 30 min prior to measuring absorbance at 450 nm using a microplate reader (Synergy HT, BioTek, USA).

### Oil red O staining

Rat corpus cavernosum endothelial cells were seeded in 12-well plates (2 × 10^5^ cells/well) and subjected to the interventions for 48 h after adhesion. Medium was aspirated and cells were washed with phosphate-buffered saline (PBS), fixed in 4% paraformaldehyde, and stained with Oil Red O solution (Solarbio, China) for 30 min. Excess dye was differentiated with 60% isopropanol. Nuclei were counterstained with hematoxylin (Beyotime, China). Cells were washed with PBS and imaged immediately under an inverted microscope (CKX41, Olympus, Japan).

### 2′,7′-Dichlorodihydrofluorescein diacetate (DCFH-DA) fluorescent probe assay

Rat corpus cavernosum endothelial cells were seeded in 12-well plates (2 × 10^5^ cells/well) and subjected to the interventions for 24 h after adhesion. Cells were incubated with DCFH-DA (Beyotime, China) in serum-free medium for 30 min at 37°C in the dark. The probe was removed and cells were washed with PBS. Nuclei were counterstained with Hoechst 33342 (Aladdin, China). Fluorescence was imaged immediately on an inverted fluorescence microscope (Ts2R, Nikon, Japan) using identical exposure settings. Intracellular ROS was quantified as the mean fluorescence intensity (MFI) of DCF per field with ImageJ software (version 1.51).

### Endothelial function assessment

In Transwell migration assay, RCECs were serum-starved using 5% fetal bovine serum (FBS)-containing ECM for 24 h prior to treatment. The lower chamber contained 500 μL 10% FBS-containing DMEM. The upper chamber received 2 × 10^5^ cells suspended in 200 μL 1% FBS-containing DMEM (± 20 μg/mL ox-LDL, ± 2 μM atorvastatin, ± 10 μM H89). After 24 h, non-migrated cells were removed from the upper surface. Migrated cells on the underside were fixed, stained with 0.1% crystal violet (Beyotime, China), imaged, and counted in ≥5 random fields/insert.

In tube formation assay, matrigel (Corning, USA) was thawed on ice and 200 μL/well was dispensed into pre-chilled 24-well plates, polymerized at 37°C for 30 min. Rat corpus cavernosum endothelial cells (1.5 × 10^5^ cells/well) were seeded in DMEM (± 20 μg/mL ox-LDL, ± 2 μM atorvastatin, ± 10 μM H89) and incubated 8 h. Tube formation was imaged and quantified using ImageJ Angiogenesis Analyzer (version 1.51).

### Immunofluorescence

Rat corpus cavernosum endothelial cells were co-incubated in the presence or in the absence of ox-LDL (20 μg/mL), atorvastatin (2 μM) or the PKA inhibitor H89 (10 μM, GlpBio, USA). After 24 h, RCECs grown on glass coverslips were fixed in 4% paraformaldehyde for 30 min. After blocking with 5% BSA for 30 min, cells were incubated overnight at 4°C with primary antibodies against phosphorylated endothelial nitric oxide synthase (p-eNOS) Ser1177 (1:200, PA5-104858, Thermo), p-eNOS Ser633 (1:200, PA5-105848, Thermo), and phosphorylated protein kinase A (p-PKA) Thr197 (1:200, #4781, CST). Subsequently, fluorescein isothiocyanate (FITC)-conjugated secondary antibodies (1:2000, ab6717, Abcam) were applied at 37°C for 2 h. Nuclei were counterstained with 4′,6-diamidino-2-phenylindole (DAPI; Beyotime, China). Coverslips were mounted with anti-fade medium after final PBS washes, and fluorescence images were acquired on an upright fluorescence microscope (DS-Qi2, Nikon, Japan) under identical exposure settings.

### Western blotting

Proteins were lysed from tissues or cells using radioimmunoprecipitation assay (RIPA) buffer containing protease and phosphatase inhibitor cocktails (Fdbio, China), followed by centrifugation and determination of protein concentration with a BCA assay (Fdbio, China). Proteins were separated on 10% sodium dodecyl sulfate–polyacrylamide gel electrophoresis (SDS-PAGE) and electrotransferred onto polyvinylidene fluoride (PVDF) membranes (Millipore, USA), which was blocked and sequentially incubated with primary antibodies against alpha-smooth muscle actin (α-SMA), collagen I, p-PKA, PKA, p-eNOS Ser1177, p-eNOS Ser633, p-eNOS Thr495, eNOS, β-actin and a secondary antibody (Anti-Rabbit, Anti-Mouse). Signals were developed with ECL substrate and captured on a Gel imaging system (ChemiDoc XRS+, Bio-Rad, USA). Band intensities were quantified with ImageJ software (version 1.51) and normalized to β-actin, phospho-proteins were additionally normalized to their total protein.

α-SMA (1:5000, 14395-1-AP), Collagen I (1:5000, 66761-1-Ig), p-eNOS Thr495 (1:1000, 28939-1-AP), β-actin (1:20000, 60008-1-Ig), HRP-conjugated Goat Anti-Rabbit IgG(H+L) (1:5000, SA00001-2), Horseradish peroxidase (HRP)-conjugated Goat Anti-Mouse IgG (1:5000, SA00001-1-A) were purchased from Proteintech. p-eNOS Ser1177 (1:1000, PA5-104858), p-eNOS Ser633 (1:1000, PA5-105848), eNOS (1:1000, PA1-037) were purchased from ThermoFisher Scientific. Protein kinase A (1:1000, #4782), p-PKA Thr197 (1:1000, #4781) were purchased from Cell Signaling Technology.

### Statistics

Normality and homogeneity of variances were assessed using the Shapiro–Wilk test and Levene’s test, respectively. Data that met the assumptions for parametric analysis were analyzed using one-way analysis of variance (ANOVA) followed by Tukey’s post hoc test for multiple comparisons. Variables that violated the assumptions of normality or homogeneity of variances were analyzed using the non-parametric Kruskal–Wallis test followed by Dunn’s multiple comparison test. Normally distributed data are presented as mean ± SD, whereas non-normally distributed data are presented as median with interquartile range. A *P* value <.05 was considered statistically significant. All statistical analyses were performed using GraphPad Prism version 10.1.0.

## Results

### Atorvastatin improves dyslipidemia and reduces oxidative stress in HLED rats

Hematoxylin and eosin staining of liver tissue showed that HLED rats developed marked hepatic steatosis compared to controls, which was markedly alleviated by atorvastatin but not by tadalafil (Figure 1A). Consistent with these histopathological findings, HLED rats exhibited significantly elevated serum levels of LDL and TC, both of which were effectively lowered by atorvastatin but unaffected by tadalafil (Figure 1B). To further characterize the metabolic phenotype, body weight was monitored throughout the experimental period. Rats in the HLED group showed a significant increase in body weight compared with the Control group, whereas atorvastatin treatment did not induce marked body-weight loss (Figure 1C), suggesting that the beneficial effects of atorvastatin were not simply attributable to nonspecific weight reduction.

**Figure 1:**
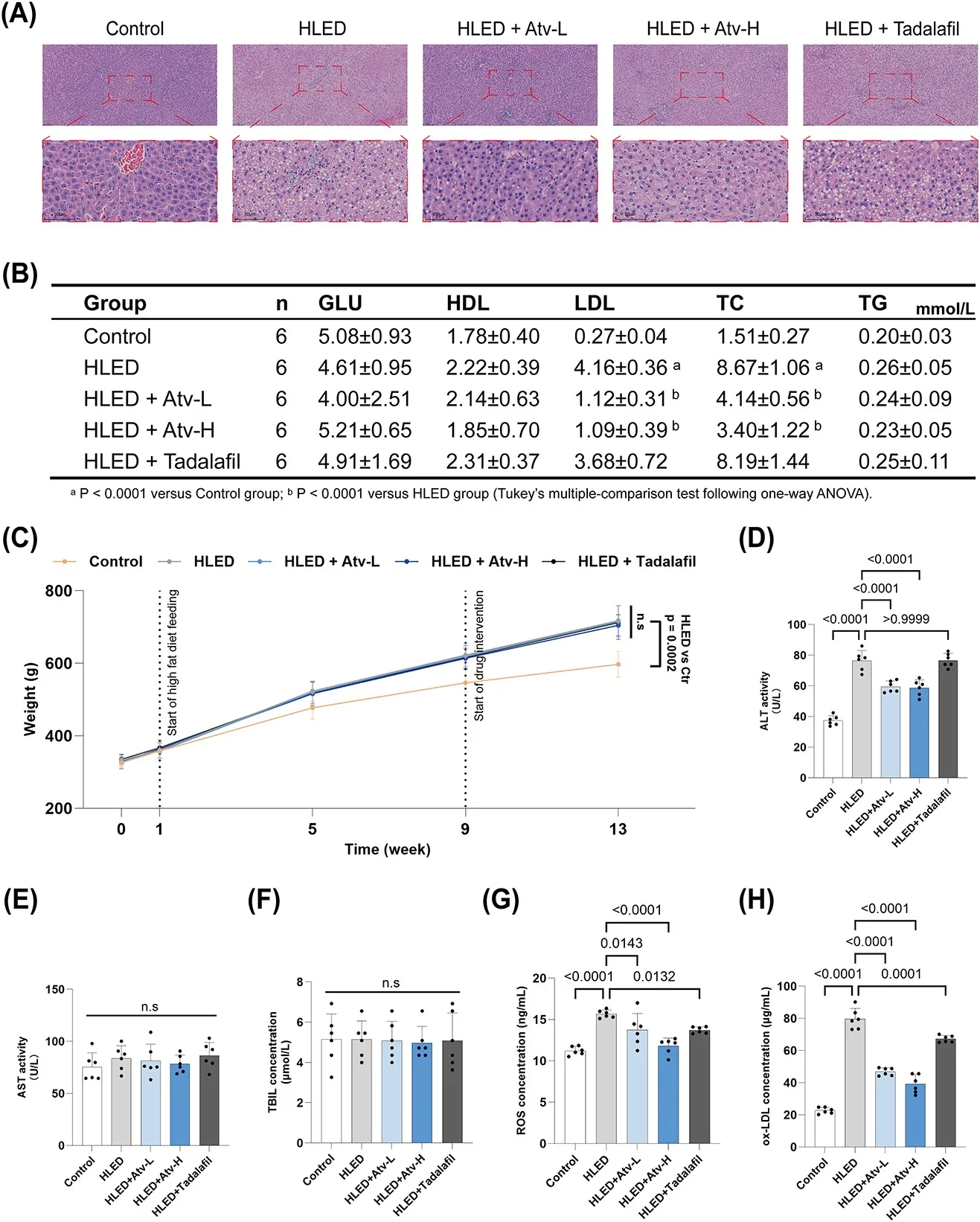
Atorvastatin’s effects on lipid metabolism and oxidative stress in HLED rats. (A) Representative liver H&E staining images from each group (*n* = 3, magnification × 10, scale bar = 250 μm), with boxes highlight areas of fatty degeneration in hepatocytes (magnification × 40; scale bar = 100 μm). (B) Serum levels of GLU, HDL, LDL, TC, and TG in each group (*n* = 6). (C) Body-weight changes in each group during the experimental period (*n* = 6). (D-F) serum levels of ALT activity, AST activity, and TBIL concentration in each group (*n* = 6). (G and H) serum levels of ROS and ox-LDL in each group (*n* = 6). Data are presented as mean ± SD. Exact *P* values are indicated in the corresponding panels. ALT, alanine aminotransferase; AST, aspartate aminotransferase; GLU, glucose; H&E, hematoxylin and eosin; HDL, high-density lipoprotein; HLED, hyperlipidemia-induced erectile dysfunction; LDL, low-density lipoprotein; ox-LDL, oxidized low-density lipoprotein; TC, total cholesterol; TG, triglyceride; ROS, reactive oxygen species.

Considering the hepatic steatosis observed by H&E staining and the potential hepatic safety concern associated with statin treatment, serum liver-function indicators were further assessed. Aminotransferase activity was significantly increased in HLED rats compared with controls, while both low-dose and high-dose atorvastatin significantly reduced ALT activity. In contrast, tadalafil did not significantly decrease ALT activity compared with the HLED group. No significant differences in AST activity or TBIL concentration were observed among the groups (Figure 1D-F). These biochemical findings were generally consistent with the histological observations, indicating that the HLED model induced hepatic steatosis accompanied by mild hepatocellular stress, whereas atorvastatin attenuated hepatic steatosis without evidence of overt liver injury under the present experimental conditions. Furthermore, systemic oxidative stress was increased in HLED rats, as indicated by higher serum ROS and ox-LDL levels. Atorvastatin treatment significantly reduced these oxidative stress markers (Figure 1G and H).

### Atorvastatin reduces penile fibrosis in HLED rats

Masson’s trichrome staining revealed marked structural fibrosis in the corpus cavernosum of HLED rats, as evidenced by increased collagen deposition and a reduced smooth muscle/collagen area ratio compared with the Control group. Atorvastatin significantly restored these histological parameters (Figure 2A-C). Consistently, Western blot analysis demonstrated that HLED rats penile tissue had an accumulation of fibrosis-related proteins, including α-SMA and Collagen I, which were also specifically reduced by atorvastatin treatment (Figure 2D-G). Notably, none of these fibrosis parameters were significantly improved in the tadalafil group.

**Figure 2:**
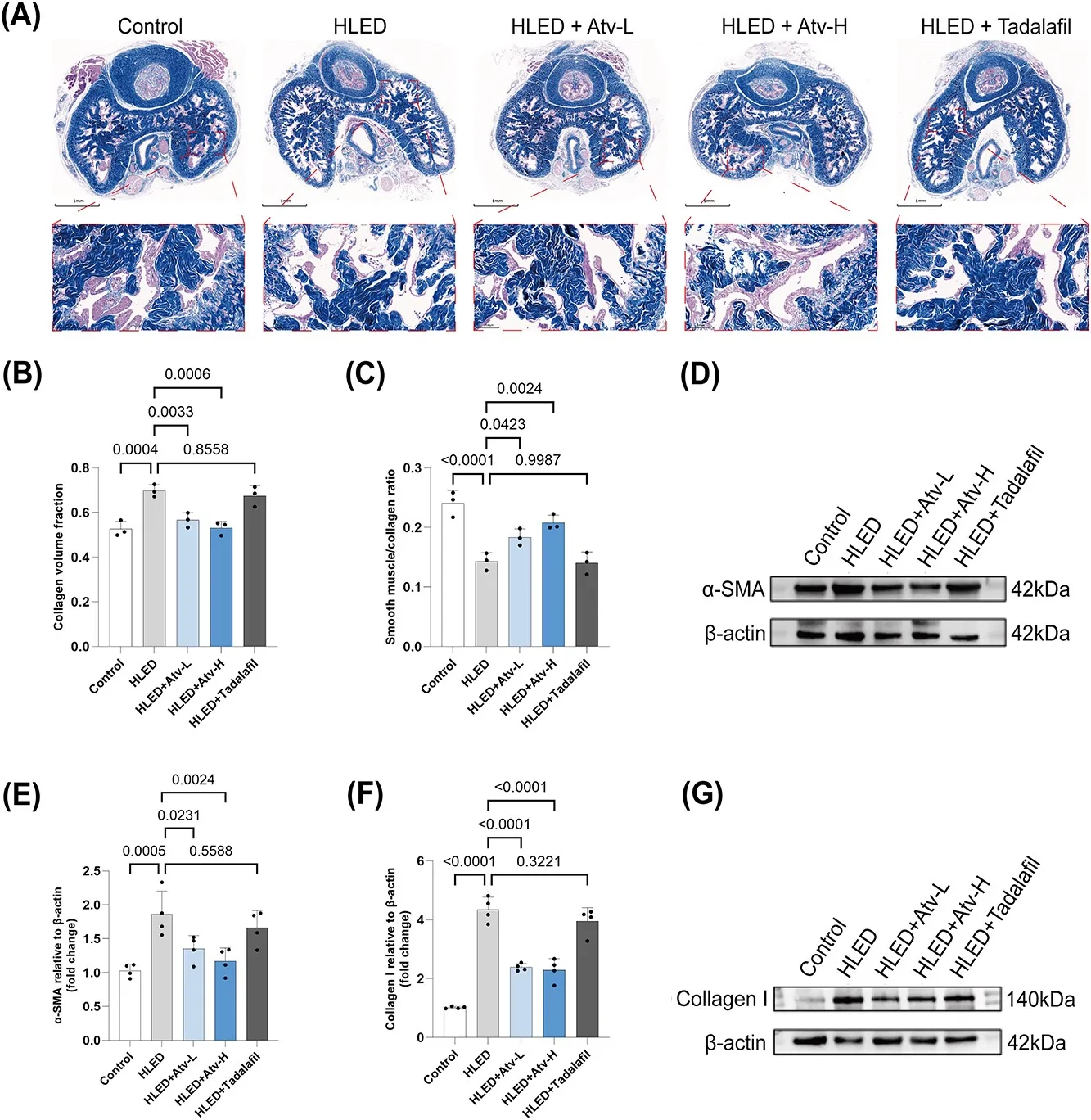
Atorvastatin’s effects on corpus cavernosum fibrosis in HLED rats. (A) Representative Masson’s trichrome staining images of penile tissue from each group (*n* = 3, magnification × 4, scale bar = 1 mm), with boxes better visualize the distribution of collagen fibers and smooth muscle bundles (magnification × 40; scale bar = 100 μm). (B and C) quantification of cavernous collagen volume fraction and smooth muscle/collagen area ratio for each group (*n* = 3). (D-G) representative western blots and corresponding quantitative analysis of α-SMA and collagen I protein levels in penile tissue from each group (*n* = 4). Data are presented as mean ± SD. Exact *P* values are indicated in the corresponding panels. HLED, hyperlipidemia-induced erectile dysfunction.

### Atorvastatin improves erectile function in HLED rats

Further erectile function assessment revealed that HLED rats exhibited a complete loss of erectile responses (Figure 3A), along with significantly reduced ICP/MAP ratios and penile cGMP levels compared to controls. Atorvastatin treatment partially restored erectile function, increasing both erection frequency and the ICPmax/MAP ratio, while also elevating penile cGMP content (Figure 3B-D). Notably, tadalafil, used as a positive control, produced the most pronounced immediate improvement in erectile function, particularly in the ICPmax/MAP ratio and penile cGMP level.

**Figure 3:**
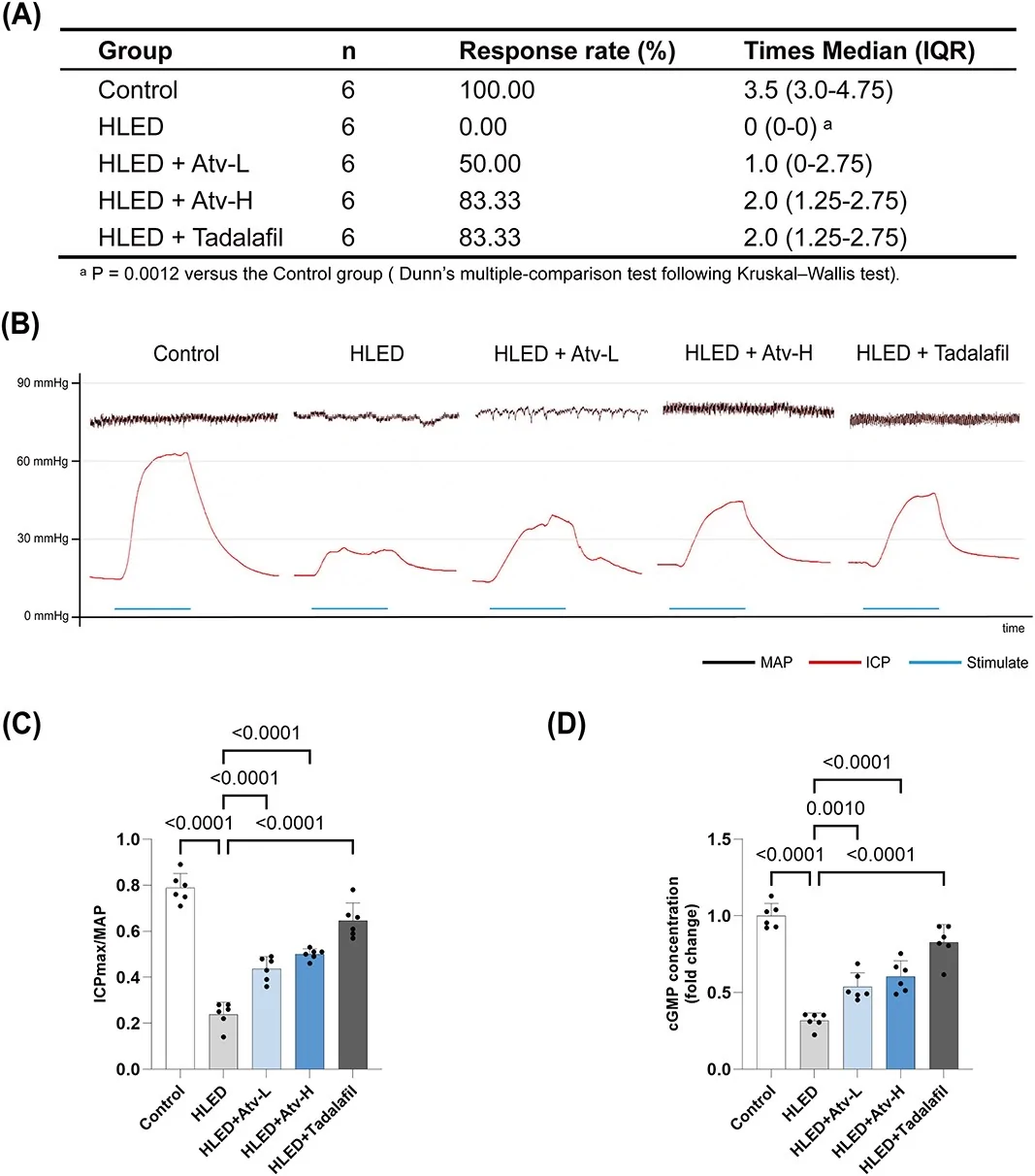
Atorvastatin’s effects on erectile function in HLED rats. (A) Frequency of APO-induced erections in each group during a 30-min observation period (*n* = 6). Response rate was defined as the percentage of rats showing at least one APO-induced erectile response. Erection frequency is presented as median (IQR) and was analyzed as discrete count data using the Kruskal–Wallis test followed by Dunn’s multiple-comparison test. (B and C) representative images and analysis of ICPmax/MAP ratio in each group (*n* = 6). (D) Penile tissue cGMP levels in each group (*n* = 6). For panels C and D, data are presented as mean ± SD. Exact *P* values are indicated in the corresponding panels. APO, apomorphine; cGMP, cyclic guanosine monophosphate; HLED, hyperlipidemia-induced erectile dysfunction; IQR, interquartile range; MAP, mean arterial pressure.

### Atorvastatin activates PKA/eNOS Ser633 pathway in vivo

We investigated eNOS activation pathways in the penile tissue of HLED rats to understand the mechanism of atorvastatin’s action. In untreated HLED rats, eNOS activity was impaired characterized by reduced phosphorylation at Ser1177 and Ser633 and elevated phosphorylation at Thr495 (Figure 4A-C). Atorvastatin treatment reversed this pattern by specifically enhancing Ser633 phosphorylation and reducing Thr495 phosphorylation, while Ser1177 phosphorylation remained unchanged (Figure 4E-G). This specific effect was associated with a marked increase in PKA phosphorylation (Figure 4D, H), indicating that atorvastatin restores eNOS function primarily via PKA- associated activation of the Ser633 site.

**Figure 4:**
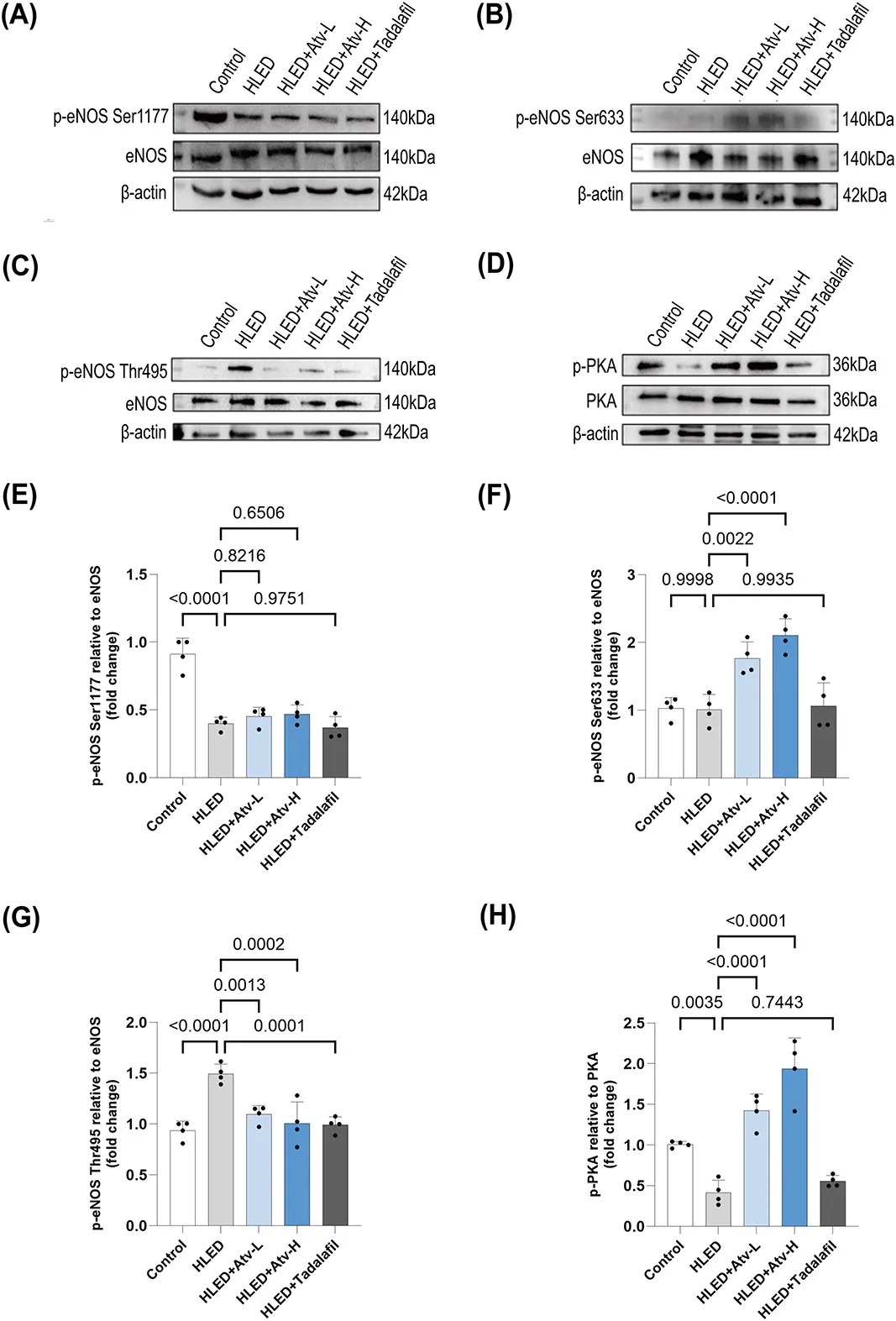
Atorvastatin’s modulation of the PKA/eNOS pathway in penile tissue. (A-D) representative western blots showing levels of p-eNOS Ser1177, p-eNOS Ser633, p-eNOS Thr495, total eNOS, p-PKA Thr197, and total PKA in penile tissue from each group (*n* = 4). (E-H) quantitative analysis of eNOS phosphorylation at Ser1177, Ser633, and Thr495 (normalized to total eNOS), and PKA Thr197 phosphorylation (normalized to total PKA) in each group (*n* = 4). Data are presented as mean ± SD. Exact *P* values are indicated in the corresponding panels. eNOS, endothelial nitric oxide synthase; PKA, protein kinase A.

### Atorvastatin ameliorates ox-LDL–induced lipotoxicity in RCECs

We next examined whether atorvastatin directly protects endothelial cells from ox-LDL–induced lipotoxic injury in vitro. Exposure to ox-LDL (20 μg/mL) significantly impaired the viability of RCECs (Figure 5B and C), promoted intracellular lipid accumulation (Figure 5D), and increased ROS production (Figure 5E and F). In contrast, co-treatment with a non-cytotoxic concentration of atorvastatin (2 μM) markedly attenuated all 3 indices of ox-LDL–induced lipotoxicity (Figure 5A-F). These in vitro findings are consistent with our in vivo observations, supporting a direct endothelial protective effect of atorvastatin under hyperlipidemic conditions.

**Figure 5:**
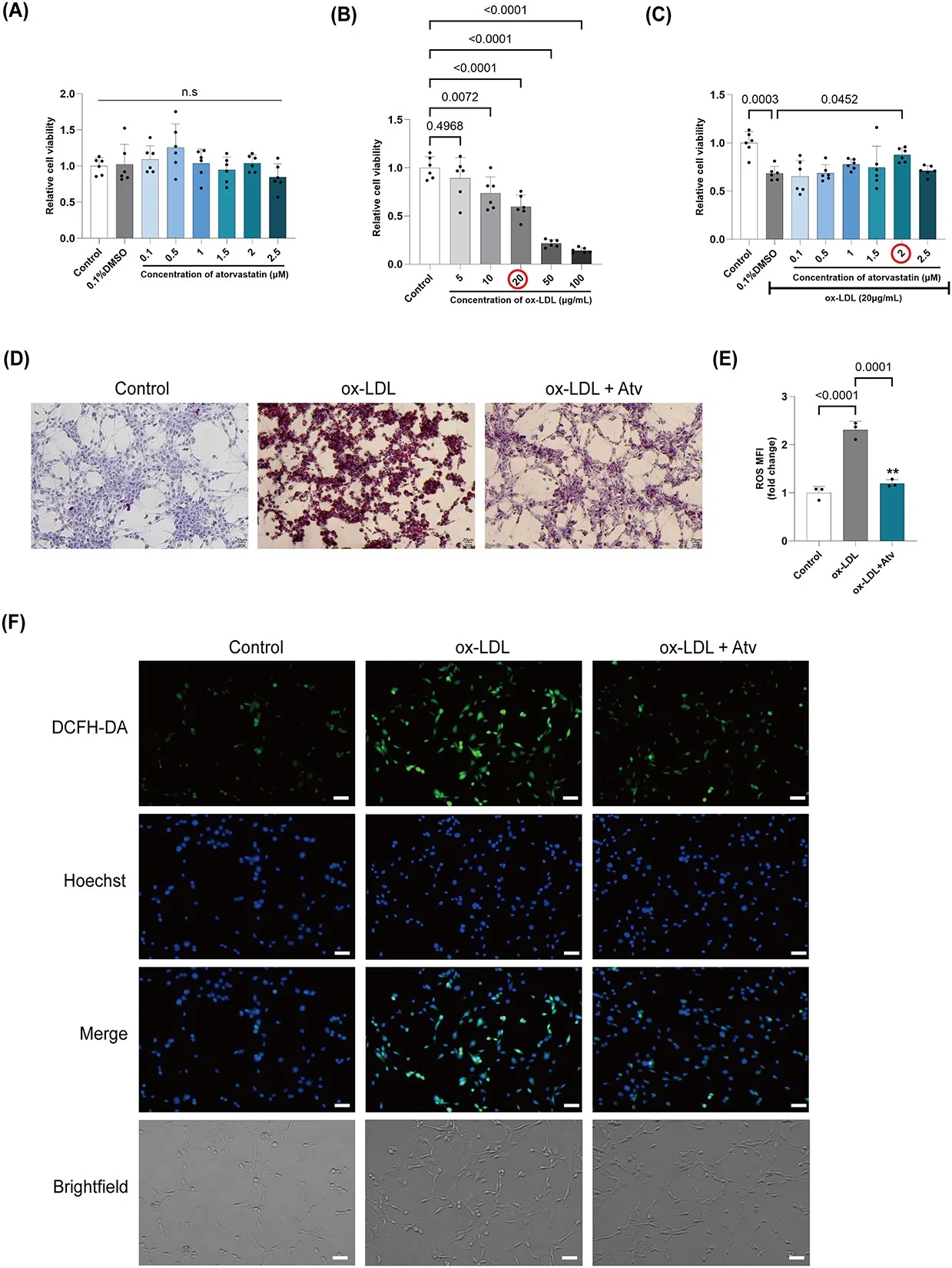
Atorvastatin’s protective effects against ox-LDL–induced lipotoxicity in RCECs. (A) RCECs viability under increasing concentrations of atorvastatin (*n* = 6). (B) RCECs viability under increasing concentrations of ox-LDL, with 20 μg/mL ox-LDL selected as the optimal concentration for subsequent modeling (*n* = 6). (C) Effects of atorvastatin on ox-LDL–induced cytotoxicity in RCECs, with the most pronounced protective effect observed at 2 μM (*n* = 6). (D) Representative oil red O staining images for each group (*n* = 3, magnification × 20, scale bar = 100 μm). (E and F) representative DCFH-DA fluorescence images of RCECs and quantitative analysis of ROS MFI for each group. Nuclei counterstained with Hoechst (*n* = 6, magnification × 20, scale bar = 50 μm). Data are presented as mean ± SD. Exact *P* values are indicated in the corresponding panels. ox-LDL, oxidized low-density lipoprotein; RCECs, rat corpus cavernosum endothelial cells; ROS, reactive oxygen species.

### Atorvastatin restores endothelial function in ox-LDL–treated RCECs

To further investigate the impact of atorvastatin on endothelial function, we performed in vitro assays using RCECs, with the PKA inhibitor H89 employed for mechanistic validation. In addition to Transwell migration, tube formation was assessed as an in vitro surrogate of endothelial angiogenic and vascular repair capacity, because coordinated capillary-like network formation reflects multiple endothelial functional processes, including extracellular matrix interaction, migration, alignment, and tubular organization. While ox-LDL significantly impaired RCEC migration in Transwell assay, co-treatment with atorvastatin restored migratory capacity (Figure 6A and B). Similarly, in tube formation assay, ox-LDL severely disrupted the formation of capillary-like networks, an effect that was significantly mitigated by atorvastatin (Figure 6A, C, D). Crucially, the PKA inhibitor H89 largely abolished the beneficial effects of atorvastatin on both migration and tube formation. In parallel, total eNOS protein level was reduced by ox-LDL compared with controls, partially restored by atorvastatin, and attenuated again in the presence of H89 (Figure 6E and F). These results suggest that atorvastatin protects endothelial function primarily through a PKA-associated pathway.

**Figure 6:**
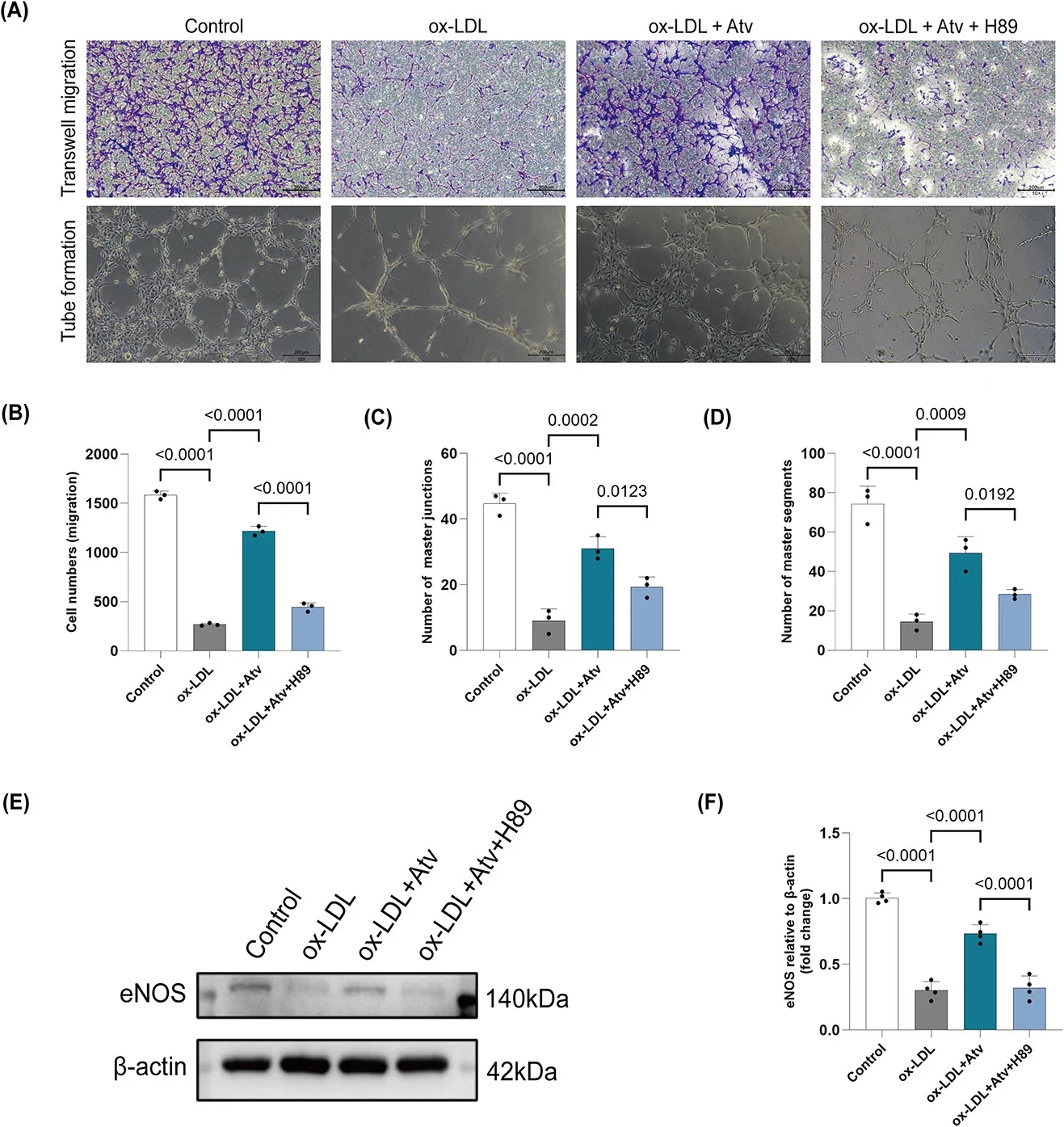
Atorvastatin’s effects on ox-LDL–induced endothelial dysfunction in RCECs. (A) Representative images of Transwell migration assay and tube formation assay for each group (*n* = 3, magnification × 10, scale bar = 200 μm). (B) Quantification of migrated cells in Transwell migration assay for each group (*n* = 3, 5 random fields per image were selected for quantification and averaged). (C and D) quantification of master junctions and segments in tube formation assay for each group (*n* = 3). (E and F) relative quantitative western blot analysis of eNOS protein level for each group (*n* = 4). Data are presented as mean ± SD. Exact *P* values are indicated in the corresponding panels. eNOS, endothelial nitric oxide synthase; ox-LDL, oxidized low-density lipoprotein; RCECs, rat corpus cavernosum endothelial cells.

### Atorvastatin promotes PKA-associated phosphorylation of eNOS at Ser633 in vitro

We further validated the mechanistic effects of atorvastatin in RCECs. Immunofluorescence analysis demonstrated that ox-LDL exposure suppressed eNOS activity, as evidenced by a marked reduction in phosphorylation at the Ser1177 site (Figure 7A and B). While Atorvastatin treatment did not restore Ser1177 phosphorylation, it significantly increased phosphorylation at the PKA-associated Ser633 site, concomitant with enhanced PKA activation. Importantly, the PKA inhibitor H89 blocked both atorvastatin-induced PKA activation and Ser633 phosphorylation (Figure 7A, C, D). These findings are consistent with our in vivo results, indicating that atorvastatin activates eNOS primarily via the PKA-Ser633 pathway under conditions of endothelial dysfunction.

**Figure 7:**
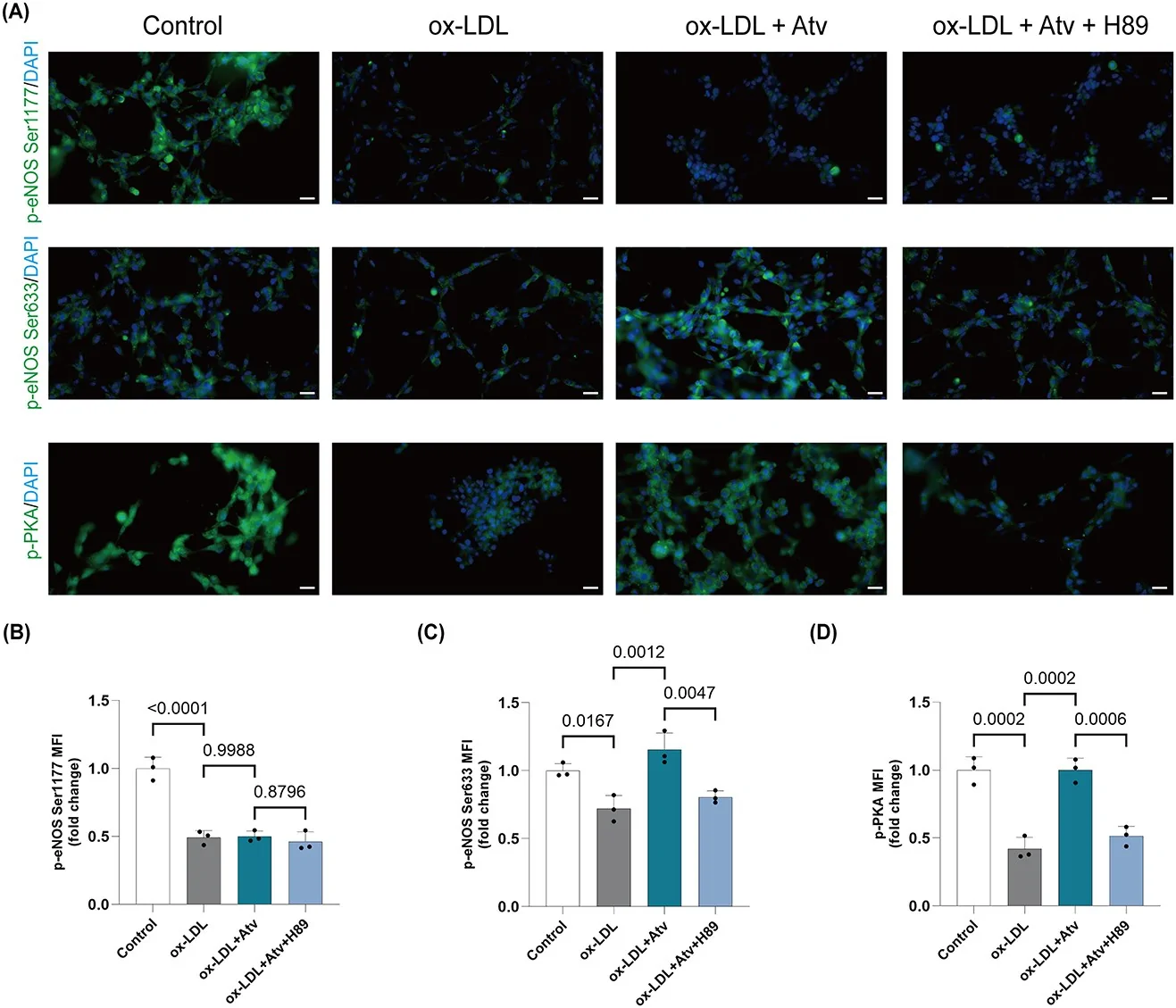
Atorvastatin’s modulation of the PKA/eNOS pathway in RCECs. (A) Representative immunofluorescence images of p-eNOS Ser1177, p-eNOS Ser633, and p-PKA Thr197 for each group. Nuclei counterstained with DAPI (*n* = 3, magnification × 40, scale bar = 20 μm). (B-D) quantification of MFI for p-eNOS Ser1177, p-eNOS Ser633, and p-PKA Thr197 for each group (*n* = 3). Data are presented as mean ± SD. Exact *P* values are indicated in the corresponding panels. eNOS, endothelial nitric oxide synthase; PKA, protein kinase A; RCECs, rat corpus cavernosum endothelial cells

## Discussion

Hyperlipidemia is a major risk factor for vasculogenic ED. In the present study, atorvastatin improved erectile function in rats with HLED, accompanied by correction of dyslipidemia, attenuation of oxidative stress, reduction of corporal fibrosis, and restoration of endothelial NO signaling. Mechanistically, these benefits were associated with increased PKA activation and preferential phosphorylation of eNOS at Ser633 in the corpus cavernosum. Taken together, our findings support the view that atorvastatin ameliorates HLED through a PKA-associated eNOS regulatory pathway.

Hyperlipidemia-induced ED is characterized by markedly reduced endothelial NO bioavailability, a hallmark of vasculogenic ED.^3^ Consistent with previous reports,^17^^,^^18^ our HLED rats exhibited reduced penile cGMP levels, increased oxidative stress, and adverse structural remodeling characterized by increased collagen deposition and loss of smooth muscle, together with markedly impaired erectile responses. These findings recapitulate key features of vasculogenic ED under chronic metabolic stress. Although this dietary model is accelerated and does not fully recapitulate the etiology of human hyperlipidemia-associated ED, it provides a reproducible platform for studying hyperlipidemia-associated vascular dysfunction and ED.^19^ Notably, atorvastatin appeared to act at both the systemic and local levels. Systemically, it improved serum lipid profiles and reduced circulating ox-LDL and ROS, changes that would be expected to relieve eNOS dysfunction.^20^ Locally, atorvastatin reduced fibrosis in the penis, preserving the smooth muscle required for erection. Our findings are consistent with previous experimental and clinical evidence showing that statin therapy preserves corporal smooth muscle structure and improves endothelial function in ED.^21^^,^^22^

A major mechanistic finding of this study is that atorvastatin was associated with preferential restoration of eNOS phosphorylation at Ser633. Ser633 is a PKA-responsive site linked to calcium-independent maintenance of eNOS activity,^11^^,^^23^ and its recovery was accompanied by increased PKA phosphorylation and reduced phosphorylation at the inhibitory Thr495 site.^24^ In vitro, H89 markedly attenuated atorvastatin-induced Ser633 phosphorylation and the associated improvement in endothelial function, supporting the involvement of a PKA-associated signaling mechanism. Given that H89 is not fully selective for PKA and may also inhibit other protein kinases, these findings should be interpreted as supportive rather than definitive evidence of PKA-specific signaling.^25^ Future studies incorporating complementary approaches, such as PKI-derived inhibitory peptides or genetic manipulation of PKA catalytic subunits, would help further validate the contribution of PKA to atorvastatin-associated eNOS Ser633 phosphorylation.

In contrast, phosphorylation of eNOS at Ser1177 was not clearly restored by atorvastatin at the examined endpoints in either in vivo or in vitro experiments. Previous studies have shown that eNOS phosphorylation sites can exhibit distinct stimulus-dependent temporal profiles.^26^ Ser1177 phosphorylation can occur rapidly and may be transient under some conditions, whereas phosphorylation of the corresponding Ser633 site generally develops more slowly. Accordingly, the absence of detectable Ser1177 restoration at the 24-h in vitro endpoint and the final in vivo endpoint does not exclude an earlier transient response.^27^^,^^28^ Importantly, however, the present endpoint-based design does not establish the complete temporal sequence or kinetics of either phosphorylation site. Thus, the preferential Ser633 phosphorylation observed here should be interpreted as an endpoint-specific signaling pattern rather than evidence that Ser633 is generally favored over Ser1177 throughout atorvastatin treatment. Time-resolved studies examining PKA activation and eNOS phosphorylation at multiple intervals will be required to define these dynamics more precisely.

Although atorvastatin significantly improved erectile function compared with untreated HLED rats, erectile parameters did not fully return to normal levels. Tadalafil produced greater improvements in the ICPmax/MAP ratio and penile cGMP level, whereas atorvastatin was additionally associated with improvements in dyslipidemia, oxidative stress, cavernosal fibrosis, and endothelial eNOS signaling. These differential effects suggest potentially complementary pharmacological mechanisms. PDE5 inhibitors enhance NO/cGMP signaling by limiting cGMP degradation, whereas statins may improve endothelial NO bioavailability through lipid-lowering and endothelial effects. Consistent with this concept, clinical evidence supports that statin pretreatment enhances the efficacy of PDE5i in patients who previously had suboptimal responses.^22^^,^^29^ Further studies are warranted to determine whether such mechanistic complementarity translates into improved treatment responsiveness in hyperlipidemia-associated vasculogenic ED.

In conclusion, this study identifies a previously underappreciated mechanism by which atorvastatin restores eNOS signaling in HLED via PKA-associated phosphorylation of eNOS at Ser633. This mechanism extends the role of statin beyond lipid lowering and reveals an alternative pathway for eNOS activation. By improving endothelial NO bioavailability, atorvastatin may complement PDE5 inhibitor-mediated amplification of NO/cGMP signaling, supporting its potential role as an adjunct therapy for vasculogenic ED in patients with hyperlipidemia. Further validation in larger experimental cohorts and clinical studies is warranted.
